# Ophthalmic In Situ Gels with Balsam Poplar Buds Extract: Formulation, Rheological Characterization, and Quality Evaluation

**DOI:** 10.3390/pharmaceutics13070953

**Published:** 2021-06-24

**Authors:** Monika Stanciauskaite, Mindaugas Marksa, Liudas Ivanauskas, Kristina Perminaite, Kristina Ramanauskiene

**Affiliations:** 1 Department of Clinical Pharmacy, Faculty of Pharmacy, Lithuanian University of Health Sciences, Sukileliai Avenue 13, LT-50161 Kaunas, Lithuania; Kristina.perminaite@lsmu.lt (K.P.); Kristina.ramanauskiene@lsmuni.lt (K.R.); 2 Department of Analytical & Toxicological Chemistry, Faculty of Pharmacy, Lithuanian University of Health Sciences, Sukileliai Avenue 13, LT-50161 Kaunas, Lithuania; mindaugas.marksa@lsmu.lt (M.M.); liudas.ivanauskas@lsmuni.lt (L.I.)

**Keywords:** balsam poplar buds, polyphenols, ocular delivery, in situ gel, cell viability

## Abstract

Balsam poplar buds are a raw material with a high content of polyphenols. Various polyphenols are known for their anti-inflammatory and antioxidant properties. In this study, an aqueous extract of balsam poplar buds was prepared in order to use environmentally friendly and non-aggressive solvents. The aqueous extract was lyophilized, and a 1% aqueous solution of lyophilized balsam poplar buds extract (L1) was prepared. L1 solution was used as a source of polyphenols for the production of ophthalmic in situ gels, so as to develop a product featuring antioxidant properties. Poloxamer 407 (P407) and hydroxypropyl methylcellulose (HPMC) were selected as gelling agents for the in situ gels. In order to select the formulations with the best conditions of use, formulations of different polymer concentrations (P407—10%, 12%, 15%; HPMC—0.5%, 0.75%) were prepared, choosing the same amount of the active polyphenol source L1. The physicochemical properties, rheological parameters, stability, and irritant effect on the rabbit corneal cell line (SIRC) were evaluated. Formulations in which P407 and HMPC concentrations were 10/0.75% and 12%/0.75% reached a gelation point close to the ocular surface temperature; the gels remained stable for 30 days and did not cause an irritant effect on the SIRC cell line.

## 1. Introduction

Eyes are the most important sensory organ, through which we get 80% of all information [1]. Globally, the number of persons who have moderate and severe eye disorders is constantly growing. Inflammatory eye diseases are among the most common diseases in contemporary societies [2]. The causative agents of these diseases are viruses, bacteria, fungi, and protozoa [3]. With the increasing popularity of natural aids, the demand for natural ophthalmic preparations is growing as well, but the use of natural materials in ophthalmology should be based on scientific research [3,4]. The data from scientific research show that rational nutrition and consumption of fruit and vegetables help to preserve vision and even reverse vision disorders [4,5]. That positive effect is associated with the presence of some phytochemicals, which have bioactive properties, such as polyphenols and carotenoids [5]. Polyphenols can diminish the frequency of diseases—neurodegenerative diseases included—associated with oxidative stress [6,7,8]. Eye drops with polyphenols are used in glaucoma treatment in order to suppress neuroinflammation, which is responsible for neurodegeneration and retinal ganglion cell (RGC) death [9,10,11]. For patients who have dry eye syndrome (DES), symptomatic treatment is applied using eye drops, which are called artificial tears [12]. Polyphenolic additives in artificial tears have antioxidative, antibacterial, and anti-inflammatory properties, which are important in seeking a positive treatment effect [13,14]. Polyphenol-enriched eye drops are effective in protecting trabecular meshwork cells from oxidative stress [15].

Eye drop compositions usually contain plant and propolis extracts [16,17]. The use of plant extracts and propolis in eye drops is associated with their antibacterial [18], anti-inflammatory [19], and antioxidative activity [20,21,22]. These medicinal properties are mostly associated with the biologically active compounds, such as anthraquinones, flavonoids, and phenolic acids [23]. Propolis collected in Europe is often defined as poplar-type propolis, because poplar buds are one of the primary sources of propolis [24]. Since ancient times, poplar buds extracts and decoctions have been used for the alleviation of dermatitis symptoms, treatment of rheumatism or infections of the upper respiratory tract, and wound healing. Balsam poplar buds, as with propolis, are a source of phenolic acids and flavonoids. Researchers have found that *p*-coumaric acid predominates in both ethanolic propolis and balsam poplar buds extracts collected in Lithuania [25]. Poplar buds, due to their antibacterial [26], antioxidant [27], and anti-inflammatory [28] action, are a potential active material in the production of eye drops. Italian scientists determined that the use of eye drops with *p*–coumaric acid protects eye tissues and lessens the harmful effect of UVB rays, due to its antioxidative properties. *p*-Coumaric acid is useful in protecting the eyes from free radical damage, which can be caused by solar rays and UV lamps. Scientific research has proven that eye nanogel with ferulic acid positively impacts the growth of fibroblasts, as well as wound healing [29,30]. The results of scientific research show that eye drops with propolis decrease the inflammation of the cornea [31] and, due to their antibacterial properties, can serve as an auxiliary measure in cases of keratitis [32]. On the basis of the scientific research data published in existing scientific literature, it is possible to declare that the search for new natural polyphenolic compounds suitable for use in ophthalmology is still ongoing. For our research we chose balsam poplar buds (*Populus balsamifera* L.), which are one of the main plant-based precursors of propolis. Therefore, this study presents new data on the application of poplar buds extract in the modelling of ophthalmological preparations. The use of balsam poplar extract in eye drops would expand the group of users, because propolis preparations are not acceptable for vegans. One of the important stages is the selection of a pharmaceutical form.

Eye drops comprise the biggest part of ophthalmic preparations. The major problems in conventional liquid ophthalmic formulations are the washing out of the drug from the precorneal area immediately upon instillation because of constant lachrymal secretion, nasolacrimal drainage, and the short precorneal residence time of the solution [33]. This problem can be overcome by using in situ gels. In situ gels are conveniently dropped as a solution into the conjunctival sac, where they undergo a transition into a gel, with favorable residence time [34]. Ocular drug delivery systems based on the concept of in situ gel formation are aimed at longer precorneal residence time, improved ocular bioavailability, and improved patient acceptability. In situ gels are a suitable alternative for common eye drops. After dropping aqueous solution—which contains temperature-sensitive polymers—into the conjunctival sac, viscous and mucoadhesive gels are formed on the surface of the eye [35]. This leads to the improvement of precorneal residence time and ocular bioavailability. Poloxamer 407 was chosen as a gelling substance [36], as it is widely used in biomedicine because of its low toxicity and compatibility with many excipients.

Poloxamer is widely used in pharmaceutical formulations as the carrier for most routes of administration, including the rectal, vaginal, ocular, intranasal, topical, and oral routes [37]. Hydroxypropyl methylcellulose (HPMC) was chosen for its ability to prolong drug release and as a viscosity enhancer [38]. HPMC has good biological compatibility, and is nontoxic to humans. The quality of gels produced in situ is evaluated by determining various parameters, such as their physical appearance, drug content, clarity, pH value, viscosity, drug release, rheological properties, behavior, and sol–gel transition temperature. It is necessary to evaluate the modeled eye drops for their possible irritant effect on the eyes, because eyes are a sensitive organ. Scientists perform these tests using animal models and ocular cell models [39]. In order to avoid harmful effects on animals, a short time exposure (STE) in vitro test using a rabbit corneal cell line (SIRC) is recommended as an alternative method for assessing eye irritation [40]. The aim of this research is to adapt the balsam poplar buds extract for use in the production of ophthalmic gels in situ, and to evaluate their quality by conducting tests of their chemical composition, rheological properties, and biological activity in vitro. Promising results of these tests can serve as a basis for further research.

## 2. Materials and Methods

### 2.1. Materials

Reagents, standards, and solvents of analytical grade were used. Purified deionized water was prepared with the Milli-Q^®^ (Millipore, Arlington, Massachusetts, USA) water purification system. For food purposes, 96.3% rectified ethanol (JSC “Vilniaus Degtine”, Vilnius, Lithuania) was used, along with Folin–Ciocalteu reagent (Sigma-Aldrich, Buchs, Switzerland); acetonitrile (Sigma-Aldrich, Steinheim, Germany); reference standards *p*-coumaric acid (Sigma-Aldrich, Steinheim, Germany), caffeic acid (Sigma-Aldrich, Steinheim, Germany), ferulic acid (Sigma-Aldrich, Buchs, Switzerland), chlorogenic acid (Sigma-Aldrich, Steinheim, Germany), vanillic acid (Sigma-Aldrich, Buchs, Switzerland), cinnamic acid (Sigma-Aldrich, Germany), apigenin (Sigma-Aldrich, Buchs, Switzerland), pinobanksin (Sigma-Aldrich, Buchs, Switzerland), pinocembrin (Sigma-Aldrich, Buchs, Switzerland), galangin (Sigma-Aldrich, Buchs, Switzerland), and salicin (Sigma-Aldrich, Buchs, Switzerland); sodium carbonate (Sigma-Aldrich, Saint-Quentin-Fallavier, France), and aluminum trichloride hexahydrate (Sigma-Aldrich, Steinheim, Germany). An ultrasonic bath (Bandelin electronic GmbH & Co.KG, Germany) and a lyophilizer (LyoQuest Telstar, Wertheim, Germany) were used for preparation of the extracts.

### 2.2. Populus Balsamifera Extraction

Balsam poplar buds were collected in Lithuania in March 2020 from the supplier Jadvyga Balvočiūtė’s organic herb farm; fresh material was dried by the supplier. Purified water was chosen as the extractant for the extraction of the balsam poplar buds. Extraction was performed in an ultrasonic bath [41] for 60 min at a temperature of 25 °C, with a 1:10 ratio of raw material to extractant. After receiving the aqueous balsam poplar buds extract (L2), the extract was freeze-dried (lyophilized) [42]. Next, 100 mL of aqueous balsam poplar buds extract was frozen, the frozen extract was placed in a lyophilizer, and freeze-drying was carried out at −50 °C for 24 h. Then, 1% aqueous solution (L1) was prepared from the freeze-dried balsam poplar buds extract powder, which was then used in experimental ophthalmic formulations. The extracts were stored in a refrigerator at 5 °C.

### 2.3. Total Phenolic Compounds

The total content of phenolic compounds was determined in an aqueous extract of balsam poplar buds, and in a 1% aqueous solution of balsam poplar buds’ lyophilized form. The reaction was performed according to the method of Singleton et al., with some modifications [43]. The phenolic compounds content was determined using the Folin–Ciocalteu reagent, with the results expressed as the *p*-coumaric acid equivalent/g of dry weight (mg CAE/g, DW). The extracts were prepared in 25 mL volumetric flasks; 1 mL of extract, 9 mL of purified water, and 1 mL of Folin–Ciocalteu reagent were added; after 3 min, 1.5 mL of Na_2_CO_3_ was also added. The reaction mixture was then diluted to the 25 mL mark with purified water. Samples were incubated for 40 min at room temperature (RT) in the dark. The absorbance was measured using a spectrophotometer (Agilent Technologies 8453 UV-Vis, Santa Clara, California, USA) at a wavelength of 760 nm.

### 2.4. Total Flavonoids

Solutions of extracts were prepared in 25 mL volumetric flasks, with 5 mL of the extracts added to a volumetric flask and diluted up to the mark with 96% ethanol (*v*/*v*). A further reaction with diluted extracts for the identification of total flavonoids was performed in a new 25 mL flask, according to Woisky and Salatino’s methodology, with some modifications [44]. Then, 1 mL of the extracts’ solution was added to the flask, followed by 10 mL of 96% ETOH (*v*/*v*) and 2 mL of AlCl_3_ (10%) added to the volumetric flask, and the reaction was carried out in an acid medium (33% acetic acid). The reaction mixture was stirred, left in the dark at RT for 20 min and, after incubation, the reaction mixture was diluted with 96% ETOH (*v*/*v*) to the 25 mL mark. The results are expressed as the mg rutin equivalent/g of dry weight (mg RE/g, DW), and the absorbance was measured with spectrophotometer on a 407 nm wavelength.

### 2.5. HPLC Analysis

The identification of the predominant active compounds was performed via high-performance liquid chromatography (HPLC) [45]. A Waters 2695 chromatographic system with a Waters 996 diode array detector and an ACE 5C18 chromatography column (250 × 4.6 mm) was used. The data were processed using Empower 2 Chromatography Data Software. The eluent system consisted of 100% acetonitrile and 1% trifluoroacetic acid. The elution program was used as presented in Table 1, with an injection volume of 10 µL, a mobile phase flow rate of 1 mL/min, a flow time of 81 min, and a column temperature of 25 °C. The active polyphenols in testing samples were identified, evaluating the retention time of the analytes and reference substances present, as well as the UV absorption from 300 to 360 nm. The reference compounds were salicin (R^2^ = 0.9999), *p*-coumaric acid (R^2^ = 0.9999), caffeic acid (R^2^ = 0.9999), vanillic acid (R^2^ = 0.9999), cinnamic acid (R^2^ = 0.9999), ferulic acid (R^2^ = 0.9999), chlorogenic acid (R^2^ = 0.9999), apigenin (R^2^ = 0.9999), galangin (R^2^ = 0.9998), pinobanksin (R^2^ = 0.9999), and pinocembrin (R^2^ = 0.9998). The extracts were diluted 10 times with 70% ethanol (*v*/*v*). The results are presented as the mean of three measurements, *p* < 0.05.

### 2.6. Ophthalmic Gel Formulation

Ophthalmic gels were formulated using different concentrations of polymers. Poloxamer 407 (Fagron, St. Paul, MN, USA), hydroxypropyl methylcellulose (HPMC) (Sigma-Aldrich, Steinheim, Germany), propane-1,2-diol (AppliChem GmbH, Darmstadt, Germany), purified water, and a 1% solution of lyophilized balsam poplar buds extract (L1) were used to form the experimental in situ gels. We chose to add 10% (*w*/*v*) of 1% solution of balsam poplar extract (L1) to each formulation. Poloxamer 407 and HPMC gels were prepared separately. The appropriate amount of poloxamer was weighed (10%, 12%, or 15% (*w*/*v*)) and mixed with the appropriate amount of purified water, and the mixtures left in a refrigerator (5 °C) for 24 h. The HPMC mixture was prepared by weighing an appropriate amount (0.5% or 0.75% (*w*/*v*)) of polymer and adding an appropriate amount of water, with the mixture placed on a magnetic stirrer at 50 °C until a homogeneous gel form was obtained. In the preparation of the in situ gels, the poloxamer 407 and HPMC gels were mixed with a magnetic stirrer to form a homogeneous structure. Next, 10% (*w*/*v*) propylene glycol and 10% (*w*/*v*) [46] L1 were added dropwise to the formulations, and the gels were mixed to form a homogeneous structure. All experimental formulations were stored in the refrigerator (5 °C).

### 2.7. In Vitro Release Test Determining Total Phenolic Compounds

Release of phenolic compounds from formulations was performed using Franz-type diffusion cells with natural cellulose dialysis membranes (Medicell International Ltd., London, UK). Phosphate-buffered saline of pH 7.4, which is most commonly used in ophthalmic release tests, was used as the acceptor medium, with a volume of 15 mL. During the release test, the temperature was maintained at 34 ± 0.5 °C, and the medium was stirred continuously [47,48]. Samples of 1 mL of acceptor medium were taken every hour; the last samples were taken after 8 h. The total amount of phenolic compounds in the acceptor medium is expressed as the *p*-coumaric acid equivalent/g of dry weight (mg CAE/g, DW).

### 2.8. Physical Characterization (pH, Viscosity)

The pH of the experimental formulations was evaluated at room temperature with a pH meter (766 with a Knick SE 104N electrode). The pH meter was calibrated with buffer solutions at pH 4.0–7.0. The viscosity of the gels was assessed with a vibrating viscometer (Vibro viscometer SV-10, A&D Company ltd, Tokyo, Japan) at 5 °C (immediately after removal from refrigerator), at room temperature, and at 37 °C (the experimental in situ gel samples were kept in a thermostat).

### 2.9. Rheological Tests

The rheological properties of the in situ formulations were evaluated with a rheometer (Physica MCR, Anton Paar GmbH, Austria) using a system of parallel steel plates and a standard-size concentric cylinder geometry, taking the formulation composition system into account. Storage modulus G′ and loss modulus G″ were measured using a system of parallel plates, the samples were carefully placed on the lower rheometer plate, and the measurements were carried out at a temperature from 20 °C to 50 °C, at an angular frequency omega of 1 rad/s, amplitude gamma of 0.5%, and temperature change rate of 2 °C per min. Flow properties were assessed using a concentric cylinder system, containing 10 g of the experimental formulation substance in a concentric cylinder; measurements were made at 22 °C, 32 °C, and 37 °C, with the shear rate from 1 to 200 1/s [36,49]. Data were processed using RheoPlus software (Anton Paar GmbH, German). The analysis was performed at least three times for each composition.

### 2.10. Cell Viability: The Short Time Exposure (STE) Test

A rabbit corneal cell line (SIRC, American Type Culture Collection (ATCC)) was used for the study. The cell line was cultivated in the flask (75 cm^2^) according to the protocol provided by the ATCC [50]. Eagle’s Minimum Essential Medium (American Type Culture Collection (ATCC)) was used, with 10% fetal bovine serum (FBS) (*v*/*v*), 1% penicillin/streptomycin solution, and 1% L-glutamine (all reagents were purchased from Life Technologies, Thermo Fisher Scientific, Waltham, MA, USA). Cells were grown in a thermostat at 37 °C with a CO_2_ level of 5%.

The STE assay was performed with an SIRC cell line based on the MTT method protocol, with some modifications [51]. Cells from the SIRC culture line were seeded in 96-well plates (1 × 104 cells/well) and incubated in a 37 °C thermostat for 24 h. The experimental in situ formulations—aqueous balsam poplar buds extract (L2), and 1% lyophilized extract aqueous solution (L1)—were incubated on the cells for a short time: 5 min and 30 min, respectively. Empty gel G0 was used as a control. Extracts with a cell viability of 70% or less were considered to have an irritant effect, while those with a cell viability of 70% or more were considered to have a non-irritant effect.

### 2.11. Antioxidant Activity by ABTS and FRAP Methods

ABTS: The antiradical activity of the extracts was determined using the ABTS assay method, with certain modifications according to Yim et al.’s methodology [52]. A stock solution of ABTS (0.0548g ABTS (Sigma-Aldrich, Oakville, ON, Canada), 50 mL purified water, and 0.0095g K_2_S_2_O_8_ (2 mmol/L) (Riedel–de Haën, Seelze, Germany) was prepared. The stock solution was kept in the dark for 16 h. The ABTS working solution was prepared by diluting a stock solution with purified water until the absorption at a wavelength of 734 nm reached 0.8 +/−0.03. 3 µL of balsam poplar buds extracts and propolis extracts were mixed with 3000 µL of ABTS working solution. All reaction mixtures were incubated at room temperature for 30 min. The absorbance of the reaction mixtures was measured spectrophotometrically at 734 nm.

FRAP: Reducing activity was assessed based on Raudonės et al.’s methodology, with some modifications [53]. A working FRAP solution was prepared from 300 mmol/L sodium acetate buffer solution (0.775g CH_3_COONa (Scharlau, Sentmenat, Spain); 4 mL glacial acetic acid, diluted to 250 mL with purified water), 10 mmol/L TPTZ solution (0.0781g TPTZ (Carl Roth, Karlsruhe, Germany); 40 mmol/L HCl-acidified purified water, in a 25 mL volumetric flask), and 20 mmol/L FeCl_3_ × 6H_2_O aqueous solution (0.1352 g FeCl_3_ × 6H_2_O (Vaseline-Fabrik Rhenania, Bonn, Germany), purified water in a 25 mL volumetric flask) with ratio of 10:1:1. Next, 10 µL of balsam poplar buds extract was mixed with 3000 µL of FRAP working solution. The samples were incubated at room temperature for 30 min in the dark. The absorbance of the reaction mixtures was measured spectrophotometrically at 593 nm.

Calibration curves were obtained from Trolox standard solutions of different concentrations. The results were expressed as the µmol Trolox equivalent per gram of tested raw material (µmol TE/g).

### 2.12. Statistical Analysis

Results are expressed as the mean and standard deviation of three measurements. For variables where normal conditions were not satisfied, a correlation was calculated based on Spearman’s correlation coefficient. Independent measurements were evaluated by a non-parametric Kruskal–Wallis test. Data were evaluated and plotted using IBM SPSS Statistics 27 (SPSS Inc., Chicago, IL, USA) and OriginPro^®^2021 (OriginLab, Northampton, MA, USA). Results were considered to be statistically significant at *p* < 0.05.

## 3. Results

### 3.1. Extraction

In the first stage of the research, the quality of aqueous extracts of poplar buds was analyzed. The results of the quality evaluation of the aqueous extract of balsam poplar buds (L2) and the aqueous solution (L1) of the 1% lyophilized extract are presented in Table 2.

The data presented in the Table 2 show a statistically significant difference between the aqueous balm poplar buds extract (L2) and the 1% lyophilized extract aqueous solution (L1) (*p* < 0.05). The L1 extract solution contained a higher content of phenolic compounds and flavonoids compared to the L2 extract. After evaluating the overall quantitative parameters of the phenolic compounds, it is important to identify the predominant biologically active compounds in the extracts.

### 3.2. HPLC Analysis

The data in Table 3 show that *p*-coumaric acid dominates in all extracts. In comparison with other active ingredients, larger amounts of cinnamic acid, caffeic acid, and pinobanksin were also found. Salicin was identified in both extracts. A statistically significantly larger quantity (*p* < 0.05) of active compounds was identified in sample L2 in comparison with extract L1. In the tested extracts, the majority of the active compounds were composed of phenolic acids. A statistically significant difference (*p* < 0.05) between the total amount of phenolic acids and the total amount of flavonoids was determined. On the basis of the test results, a 1% lyophilized extract was chosen for further research.

### 3.3. In Situ Gel Formulation

During the study, six different formulations of gelled solutions containing different amounts of polymers (poloxamer 407 and HPMC) were prepared. The simulated in situ gel compositions are presented in Table 4.

**Figure 1:**
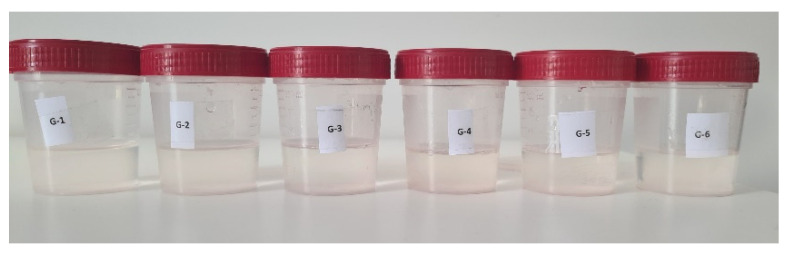
G1-G6 in situ gels with lyophilized aqueous extract of balsamic poplar buds 1% aqueous solution.

All experimental in situ gel systems had clear, colorless structure and liquid consistency at room temperature (Figure 1).

### 3.4. In Situ Gels’ Physicochemical Parameters

In order to formulate safe, non-irritant ocular gels, the physicochemical parameters such as pH, viscosity, and refractive index must be measured and evaluated. Table 5 presents the physicochemical parameters and phenolic compound contents of the prepared gels. In order to evaluate the stability of the tested formulations, the measurements of physicochemical parameters were conducted 30 days after the production of the formulations and their primary evaluation. On the basis of the results obtained, we can state that the formulations remained stable when tested immediately after the production and for 30 days after, when kept in the refrigerator. There was no statistically significant difference between them (*p* > 0.05).

The theoretical total amount of phenolic compounds in the formulations was 13.72–14.97 mg CAE/g, (standard deviation ±0.63), evaluating the total amount of phenolic c ompounds obtained via spectrophotometric methods in 1% aqueous solution. Produced in situ test formulations had a fluctuating amount of phenolic compounds, from 13.60 ± 0.64 to 14.11 ± 0.69 mg CAE/g; the sample mean was 13.90 mg CAE/g, and the standard sample deviation was ±0.23. In view of the predicted theoretical amounts of phenolic compounds in the formulations, the tested formulations are consistent with the theoretical hypothesis.

All of the produced formulations had a pH value interval in the 6.07–6.51 range. The refractive index of all of the formulations was found to be in the range of 1.355–1.360.

**Figure 2:**
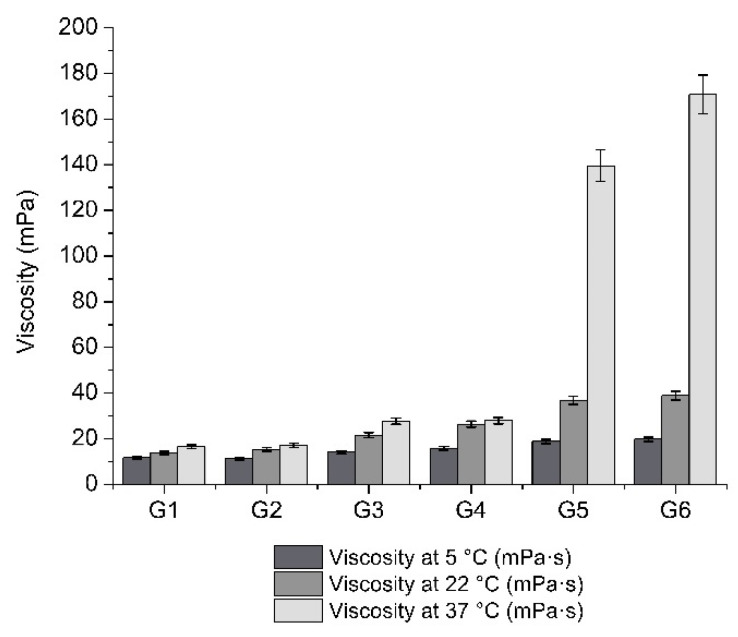
Viscosity parameters of formulations G1–G6 at 5 °C, 22 °C, and 37 °C (mean mPa·s ± SD, *n* = 3).

In situ gels are sensitive to temperature changes. The results of viscosimetric examination (Figure 2) showed that, when the temperature increased by a statistically significant degree (*p* < 0.05), the viscosity of formulations G1–G6also increased. The parameters of viscosity also showed that viscosity increased by a statistically significant amount (*p* < 0.05) when the concentration of gelating polymers in the formulations increased. The viscosity of the formulations was directly dependent on the amounts of polymers and on the temperature.

### 3.5. In Situ Gels’ HPLC Analysis

In all produced in situ gels, the same active compounds that dominated in balsam poplar water extract (L2) and in lyophilized 1% solution (L1) were identified (Table 6). Phenolic acids were also identified in ascending sequence: *p*-coumaric acid, cinnamic acid, caffeic acid, and chlorogenic acid. Flavonoids were also identified in ascending sequence: pinobanksin, pinocembrin, apigenin, and galangin. It is possible to state that the inserted active material was mostly evenly spread, because the quantity of active compounds in formulations G1–G6 was not significantly different (*p* > 0.05).

### 3.6. Rheological Characteristics

**Figure 3:**
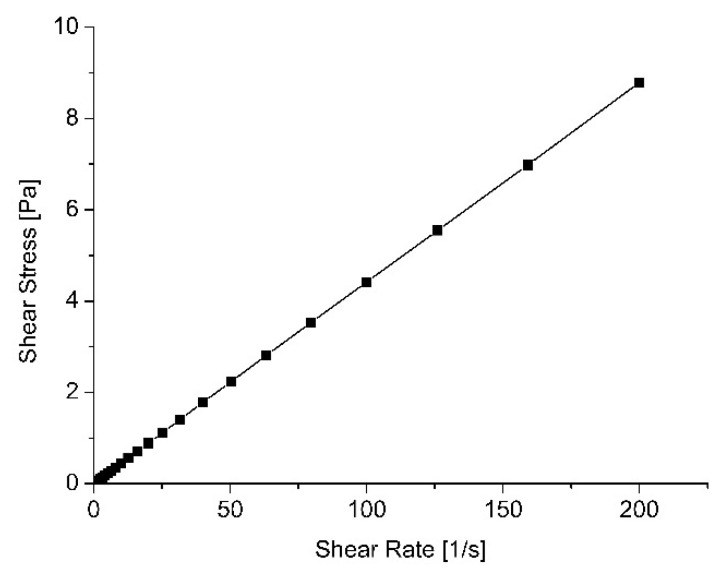
Flow curve for in situ gels G1–G6 (shear rate vs. shear stress); all formulations showed the same parameters.

Based on the shear rate (1/s) and the shear stress (Pa), a rheological flow curve can be formed, which can be used to evaluate the characteristic properties of experimental formulations G1–G6. According to the obtained rheological parameters and rheological curve, when the shear rate is controlled, all investigated formulations (G1–G6) can be assigned to Newtonian materials; the measured viscosity is independent of the shear rate (Figure 3) [54]. Because formulations G1–G6 have the properties of Newtonian materials, the consistency coefficient ((Pa·s)n) coincides with the dynamic viscosity (Pa·s).

**Figure 4:**
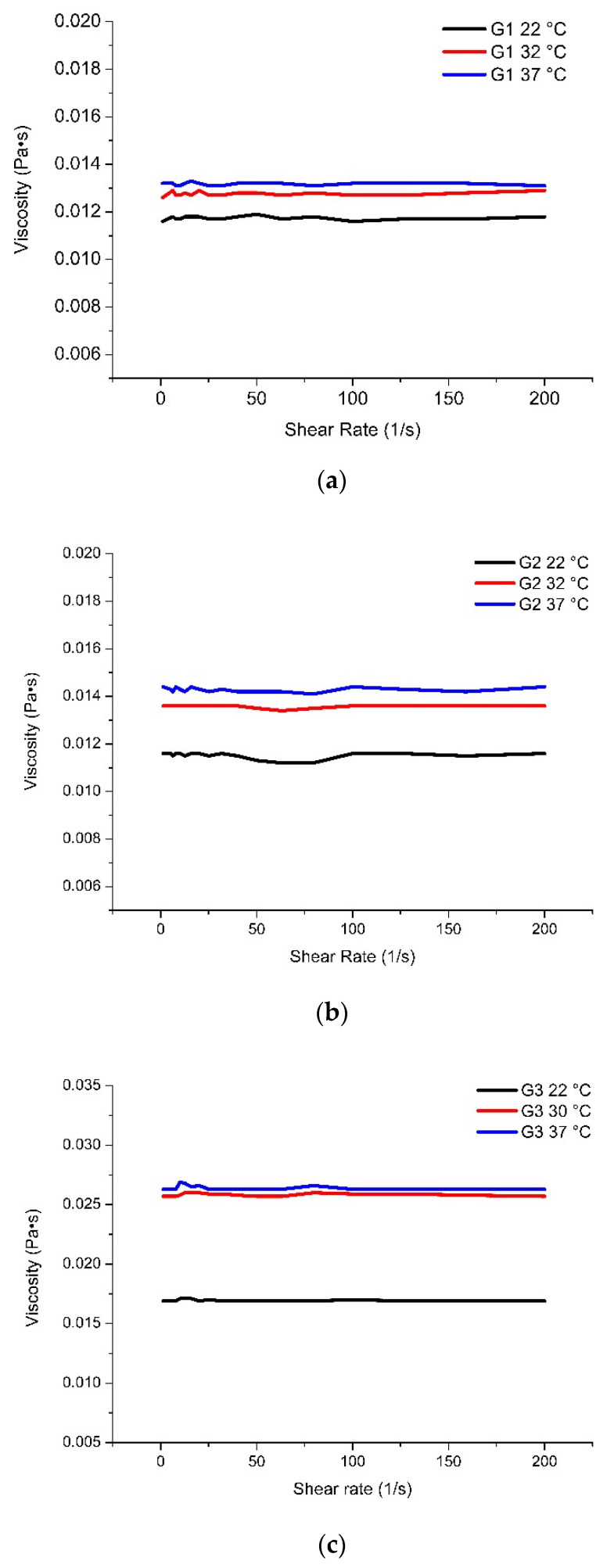

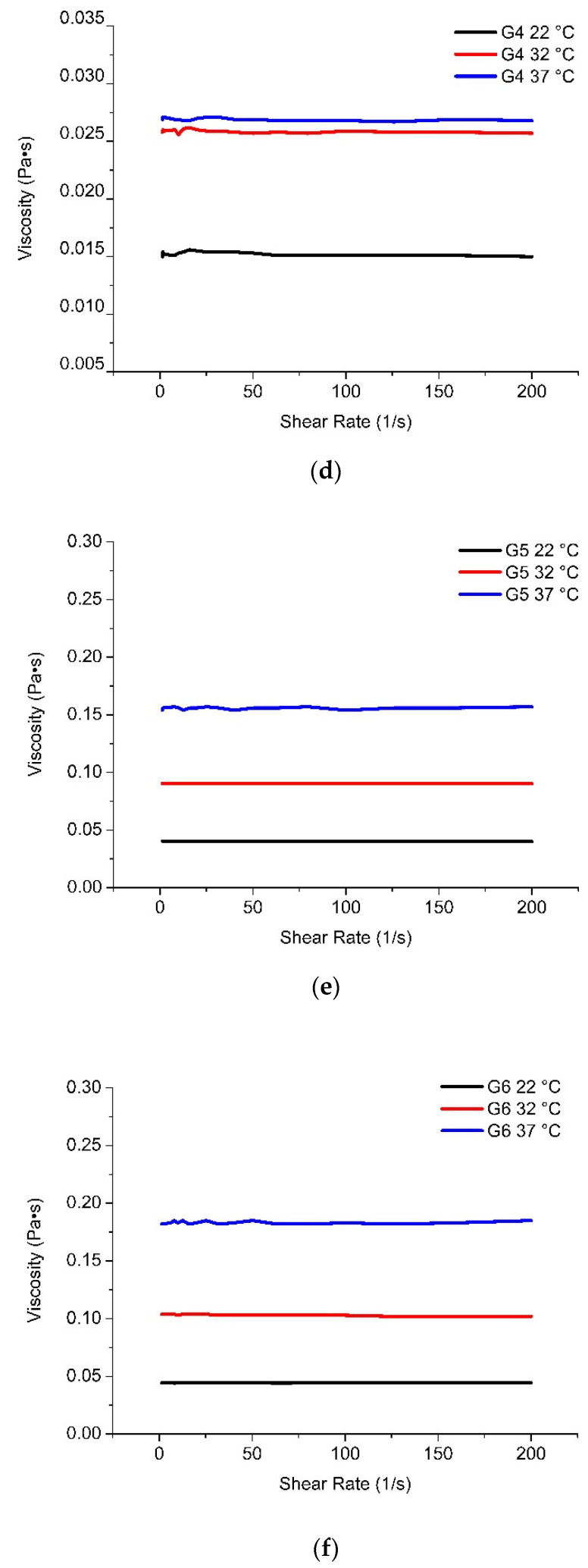
The dynamic viscosity (Pa·s) vs. shear rate (1/s) in different (22 °C, 32 °C, and 37 °C) temperatures of in situ gels: (**a**) G1; (**b**) G2; (**c**) G3; (**d**) G4; (**e**) G5; and (**f**) G6.

The formulations produced differed in their concentrations of polymers. In order to evaluate the physical properties of in situ gels, so as to customize them for ophthalmic preparations, it is important to perform rheological studies in order to assess the dynamic viscosity of the materials at important critical temperatures: room temperature (22 °C), 32 °C (the temperature of eye tissue), and 37 °C (close to human body temperature). The selected temperatures allow the condition of the products to be assessed during storage and inversion. According to the obtained rheological parameters (Figure 4), a direct correlation between the polymer concentrations in the investigated formulations and the differences in dynamic viscosity at different temperatures can be seen. At the lowest amounts of poloxamer 407 and HPMC in the formulation G1, the smallest difference in dynamic viscosity was observed at different temperatures. As the amount of HPMC increased, a greater change in dynamic viscosity was observed in gel G2 between room temperature (22 °C) and 37 °C (Figure 4a,b). Increased amounts of poloxamer 407 in gels G3 and G4—from 10% to 12%—showed a higher gel viscosity compared to gels G1 and G2. Gels G3 and G4 showed a statistically significant difference in dynamic viscosity between 22 °C and 32 °C (*p* < 0.05), while there was no statistically significant difference in viscosity between 32 °C and 37 °C (*p* > 0.05). Comparing formulations G3 and G4, a higher dynamic viscosity of formulation G4 was observed, which was affected by the higher concentration of the polymer HPMC in formulation G4 than in G3 (Figure 4c,d). Formulations G5 and G6 showed a statistically significant difference in dynamic viscosity (*p* < 0.05) at different temperatures (22 °C, 32 °C, and 37 °C). At higher concentrations of poloxamer 407 (15%), the dynamic viscosity of gels G5 and G6 differed statistically significantly from formulations G1–G4 at all temperatures. No statistically significant (*p* > 0.05) difference in dynamic viscosity compared to formulation G5 was observed with the increased HPMC concentration in formulation G6. It can be seen from Figure 4 that the dynamic viscosity of all formulations remained constant at all temperatures.

**Figure 5:**
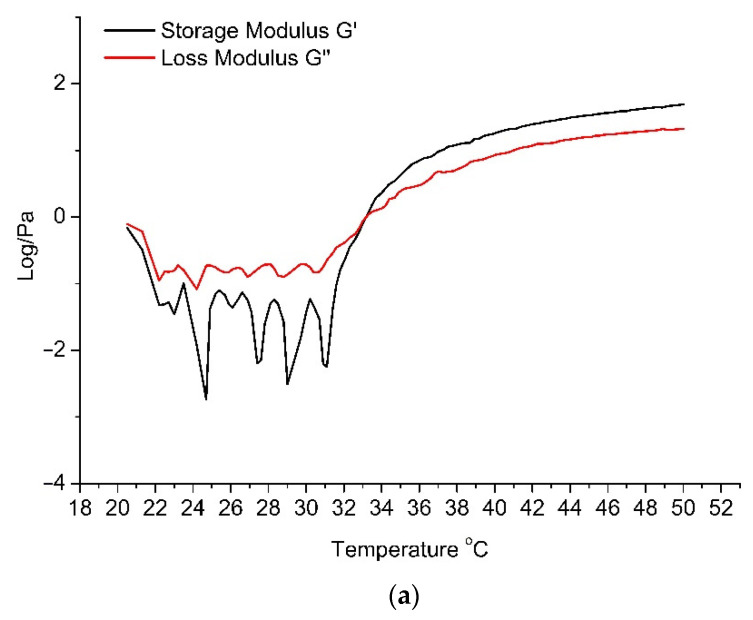

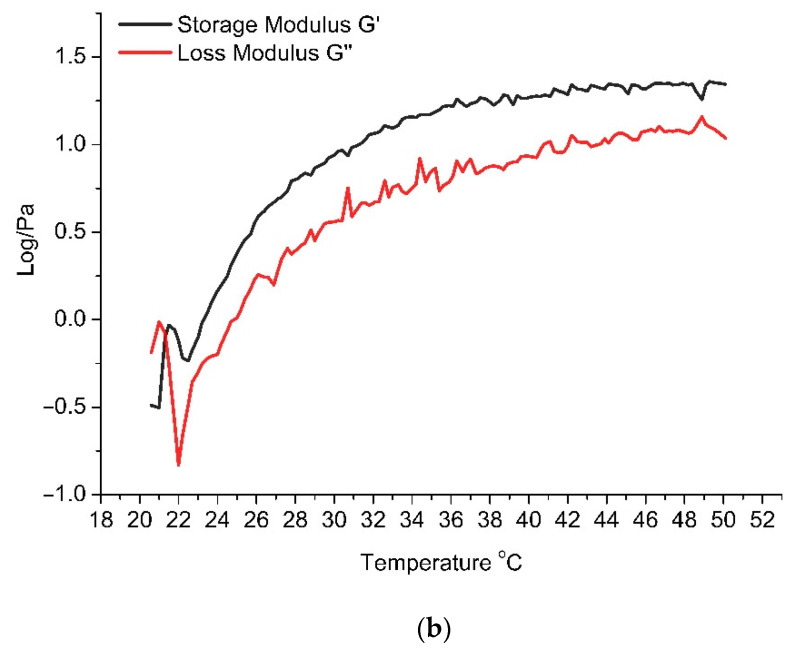
In situ gels’ gelation temperatures (storage modulus(G′), loss modulus (G″)). (**a**) G2 gel; (**b**) G6 gel.

Storage modulus G′ shows the elastic properties of the system—the energy that is stored in the material during deformation. The loss modulus G″ indicates the flow properties of the system under study and the energy dissipated. The gelation points of formulations G1–G6 were evaluated in the temperature range from 20 °C to 50 °C (Table 7). The obtained results show that gel G1 has the highest gelation point temperature—the sol-to-gel phase is reached only at 43.2 °C. However, formulation G2 (Figure 5a)—in which the concentration of poloxamer 407 is the same but the concentration of the polymer HPMC is higher—has a statistically significantly lower (*p* < 0.05) sol-to-gel temperature (33.3 °C). The sol-to-gel temperature of formulations G3 and G4 also differed statistically significantly (*p* < 0.05) at higher HPMC polymer concentrations. The lowest gelation temperature was determined in formulations G5 and G6 (Figure 5b), when the concentration of the polymer poloxamer 407 was the highest. No statistically significant difference (*p* < 0.05) was found between the gelation points of formulations G2 and G4.

Rheological studies have been performed in order to predict which of the experimental formulations would be the best suited as ophthalmic preparations.

### 3.7. In Vitro Release Studies

**Figure 6:**
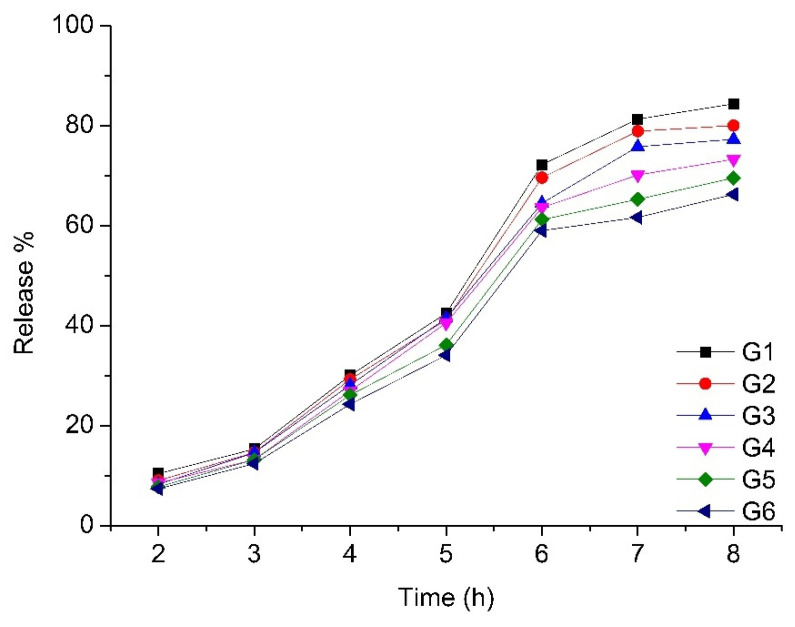
Percentage release of total phenolic compounds from experimental in situ gels G1–G6 after 8 h (mean ± SD, n = 3).

After evaluating the composition of the experimental in situ formulations via HPLC analysis, the next important step was to evaluate the release of phenolic compounds from formulations G1–G6 (Figure 6). The highest amount of phenolic compounds (mg CAE/g) was released by formulation G1 (84.39%), which had the lowest polymer concentration. The least phenolic compounds were released by the formulation G6, which had the highest concentration of polymers. Assessing the results of the release studies in vitro, a statistically significant (*p* < 0.05) difference in the amount of released phenolic compounds was observed between formulations G1 and G3, G1 and G4, G1 and G5, G1 and G6, G2 and G4, G2 and G5, G2 and G6, G3 and G5, G3 and G6, and G4 and G6. There was no statistically significant difference (*p* > 0.05) between formulations G1 and G2, G2 and G3, G3 and G4, G4 and G5, or G5 and G6. Comparing the results, a trend was observed: as the concentration of polymers in the formulation increased, the release efficiency of phenolic compounds decreased, from the very beginning of the study.

**Figure 7:**
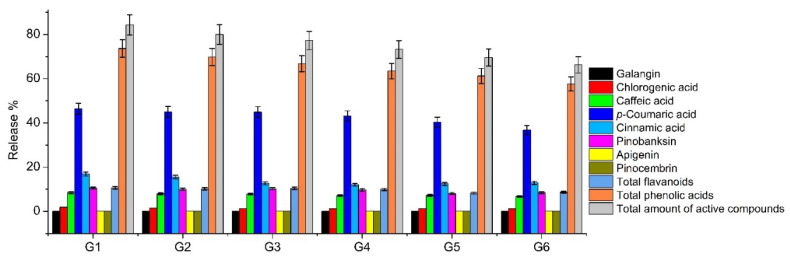
Percentage amounts of active compounds released after 8 h in experimental in situ gels G1–G6 (mean ± SD, n = 3).

From the total amount of phenolic compounds released after 8 h, the percentage released by the predominant biologically active compound formulations was estimated (Figure 7). Based on the data of the release study, of the total amount of phenolic compounds in the formulations, the predominant phenolic acid was *p*-coumaric acid. Pinobanksin was the predominant flavonoid released from the total amount of phenolic compounds after 8 h. A statistically significant difference (*p* < 0.05) was found between the amount of released phenolic acids and flavonoids. In all of the experimental formulations, the highest amount of *p*-coumaric acid and the lowest amount of galangin were released. The study showed that all of the active compounds that were identified in the prepared extracts via HPLC were released.

### 3.8. Antioxidant Activity

**Figure 8:**
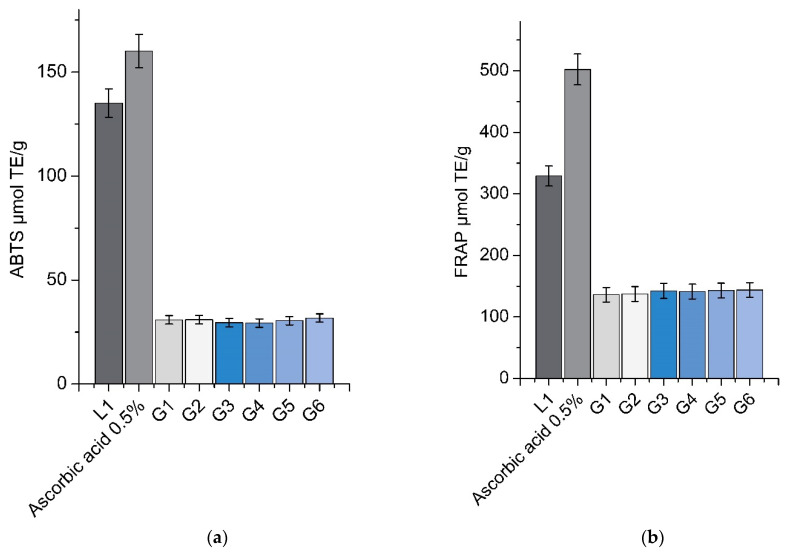
Antioxidant activity detected in in situ gels G1–G6 and lyophilized 1% aqueous solution of balsam poplar buds extract (L1) by (**a**) the ABTS method; and (**b**) the FRAP method (mean ± SD, n = 3).

Figure 8 shows the antioxidant activity of L1 (lyophilized 1% aqueous solution of balsam poplar buds extract) and in situ gels (G1–G6)—assessed via ABTS and FRAP methods—using 0.5% ascorbic acid solution for comparison. Balsam poplar buds have anti-inflammatory and antioxidant effects due to their high content of phenolic compounds. The lyophilized 1% aqueous solution of balsam poplar buds showed lower antioxidant activity compared to the 0.5% solution of ascorbic acid, using both the ABTS and FRAP methods. A statistically significant (*p* < 0.05) increase in antioxidant activity was observed in the 1% aqueous solution of lyophilized extract (L1) compared to the G1–G6 formulations, using both the ABTS and FRAP antioxidant assays; however, this was due to the lower total content of phenolic compounds in the experimental formulations. There was no statistically significant difference (*p* > 0.05) in antioxidant activity between formulations G1–G6 detected by the ABTS and FRAP methods. It can be concluded that the antioxidant activity is not affected by the different polymer concentrations present in the formulations.

### 3.9. Cell Viability: Short Time Exposure (STE) Test

**Figure 9:**
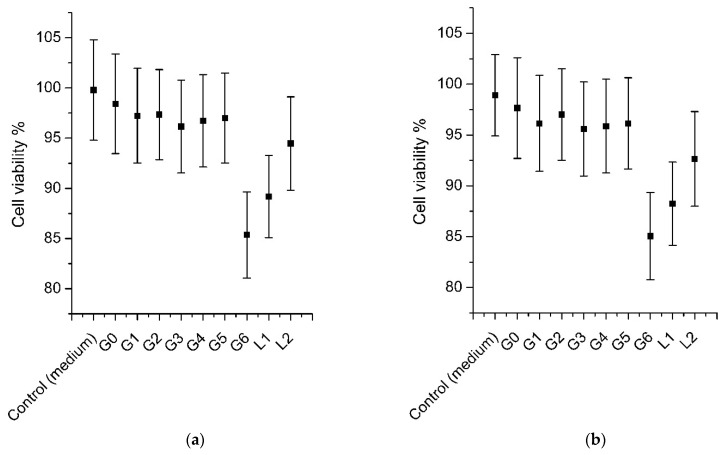
Percentage cell viability after the short time exposure (STE) test on the SIRC cell culture line (results expressed as the standard deviation of five measurements): (**a**) cell viability after 5 min; (**b**) cell viability after 30 min (mean ± SD, n = 3).

The experimental formulations were intended for the eyes; therefore, we evaluated the effects of short-term exposure of the SIRC cell line to the experimental in situ gel formulations G1–G6 and the balsam poplar buds extracts L1 and L2, using the MTT method, when the cells were exposed to the test samples for 5 and 30 min. Each well with an SIRC cell line contained 100 µL/well of the sample (Figure 9). There was no statistically significant difference (*p* > 0.05) in cell viability between formulations G1–G5. A statistically significant difference (*p* < 0.05) in cell viability was observed between gels G1–G5 and gel G6. There was a statistically significant difference (*p* < 0.05) in cell viability between extract L2 and gels G1–G5, but there was no statistically significant difference (*p* > 0.05) between extract L1 and gels G1–G5. No statistically significant difference (*p* > 0.05) was observed between extracts L1 and L2. During the study, none of the samples showed a statistically significant (*p* > 0.05) irritant effect on the SIRC cell line after 5 min or 30 min of incubation. As a result, the experimental formulations can be used as in situ eye drops.

### 3.10. Statistical Analysis

**Figure 10:**
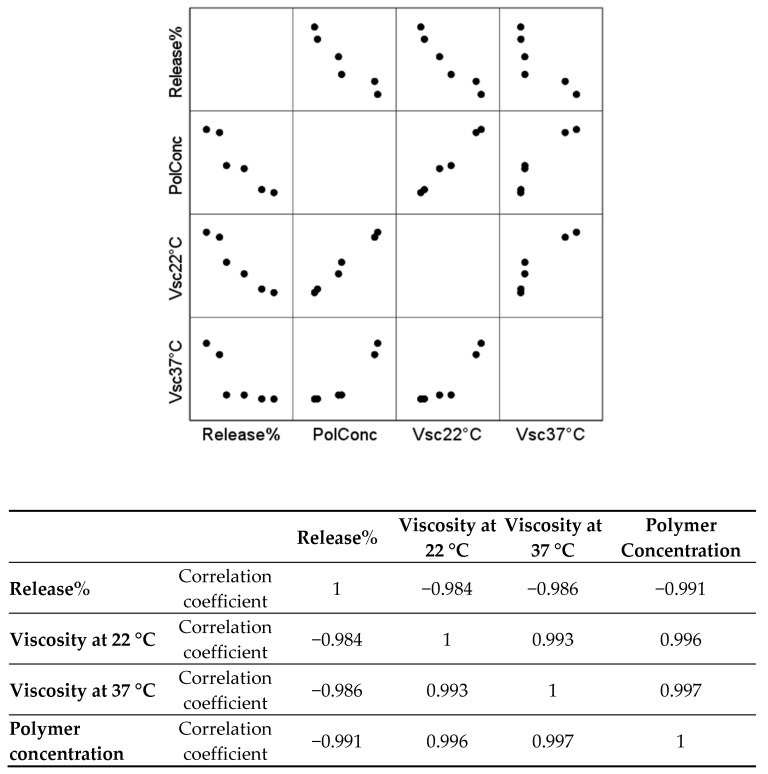
Correlation graph of total percentage release of phenolic compounds, viscosity at 22 °C, viscosity at 37 °C, and total polymer concentration (*w*/*v*), as measured by Spearman’s rank correlation coefficient.

The results of the total percentage release of phenolic compounds, viscosity at 22 °C, viscosity at 37 °C, and total polymer concentration (*w*/*v*) were evaluated according to Spearman’s rank correlation coefficient. The correlation graph presented in Figure 10 shows a strong correlation between the total percentage release of phenolic compounds, viscosity at 22 °C (ρ = −0.984), viscosity at 37 °C (ρ = −0.986), and total polymer concentration (*w*/*v*) (ρ = −0.991). Moreover, there is a strong correlation between the total polymer concentration (*w*/*v*), the viscosity at 22 °C (ρ = 0.996), and the viscosity at 37 °C (ρ = 0.997).

## 4. Discussion

### 4.1. Populus Balsamifera Extraction and Bioactive Compounds

Ultrasound-assisted extraction was applied for the extraction of the active compounds. The mechanical effects of ultrasound accelerate the process of extraction, and the use of organic solvents often becomes unnecessary. Therefore, for ultrasound-assisted extraction, water is often used as a solvent—which is an inexpensive, not dangerous, and easily accessible ecological material [41]. Balsam poplar water extracts were lyophilized in order to concentrate the resulting bioactive compounds [55]. This research confirmed that lyophilization is an effective method for the production of dry extracts with larger amounts of active compounds. Active compounds in the produced extracts L1 and L2 were identified using chromatography. The research results showed that the chosen method for the production of dry extract was suitable, since the same active compounds were identified in both extracts. The research results confirmed that lyophilization allowed us to concentrate the active compounds [56], since a larger amount of them was identified in lyophilized extract L1 (1% lyophilized water L2 extract solution) in comparison with water extract L2. The results of chemical analysis show that poplar buds extract is a potential material for the production of eye drops. *p*-Coumaric acid dominates in poplar buds extracts. *p*-Coumaric acid is a phenolic acid, which has many biological functions—such as antioxidant, anti-inflammatory, antidiabetic, antiulcer, antiplatelet, and anticancer activities [57,58]. The scientific research determined that eye drops that contained *p*-coumaric acid, due to their antioxidant properties, protected eye tissues and decreased the harmful effects of UVB rays [30]. Italian scientists determined that hydrocaffeic acid in combination with *p*-coumaric acid showed potential as a topical eye treatment against UVB damage [59]. In poplar buds extracts, the same phenolic acids were identified as in propolis. Propolis, due to its antioxidant, anti-inflammatory, and antibacterial properties, can be applied in the treatment of keratitis and corneal wounds [31].

### 4.2. Ophthalmic Gel Formulation

Poplar buds are one of the main sources of propolis; therefore, we chose that raw material for our research. They are a suitable alternative to propolis, because the raw material is more easily obtained, and is acceptable to vegans. In view of the fact that the bioavailability of eye drops can be improved by increased precorneal residence time [60], in situ gels were chosen for production. In situ gels, due to their viscosity, slow down the speed of drug elimination from the eye [46]. An important step in the formulation of in situ gels is the choice of excipients, which could ensure the stability of preparations without changing the activity of bioactive ingredients, and be non-irritant—especially when used locally on the surface of the eye [61,62]. Poloxamer 407, chosen for the production of gels, is often used for ophthalmic preparations as a substance resistant to temperature changes [63,64]. Poloxamer 407 is used for the production of in situ gels, but used alone it has few mucoadhesive properties, while too large a concentration of it can cause hyperglycemia in the eye [46,65]. In order to avoid these negative properties, poloxamer 407 is combined with cellulose derivatives, such as HPMC, usually used from 0.5 to 3% (*w*/*v*) [35,66]. This way, the poloxamer 407 concentration in the formulation is reduced, which allows control of the in situ gel’s sol-to-gel formation point. Having reached suitable concentrations of polymers, suitable gelation temperatures for ophthalmic in situ gel preparations can also be reached. Propylene glycol was used in eye drop compositions based on the work of Kurniawansyah et al. [46]. The scientific literature describes how substances such as sodium chloride or sodium dihydrogen phosphate lower gelation temperatures by forming strong crosslinks with poloxamer, while other substances such as ethanol or propylene glycol, with weaker hydrogen bonds, may weaken bonds and can increase gelation temperatures [67]. Therefore, polypropylene glycol was chosen for use to control the viscosity of in situ gels, as a co-surfactant and humectant [68]. In this study, propylene glycol content did not show an adverse effect on the physicochemical properties or biological activity of the formulations.

### 4.3. Physical Characterization of In Situ Gels

The normal physiological pH of the eyes ranges from 7 to 7.4 [69]. The formulations with poplar buds extract that we produced had pH levels ranging from 6.07 to 6.7. In eye drops, the closer the pH is to the physiological pH, the better the preparation is tolerated by the eye. Ophthalmic preparations with a pH below 4 or above 10 cause irritation and intense lacrimation, especially when the pH is strongly alkaline [69]. The pH of the produced in situ gels was in acceptable boundaries, close to the normal physiological pH. The refractive index of the produced in situ gels was between 1.355 and 1.360. The human tear film–cornea refractive index value varies from 1.335 to 1.4391 [70]. This means that the produced gels should not cause any negative reactions or eye disorders when used. Viscosity is another important parameter when evaluating the effectiveness of the eye formulation, because low viscosity allows the patient to better tolerate the preparation, and causes less discomfort when blinking. However, the smaller the viscosity, the shorter the precorneal residence time [35]. Human tears have a viscosity of about 1.5 mPa·s [71], while the recommended viscosity of ophthalmic drug formulations is within the range of 15–30 mPa·s [72]. The viscosity of in situ gels may vary from 5 to 100 mPa·s [73]. The data of our research showed that the viscosity of the produced gels varied from 11.3 to 170.8 mPa·s. It is possible to state that the viscosity of the produced in situ gels G1–G4 was in acceptable boundaries for eye formulations. Formulations G5 and G6 had higher viscosity than recommended for in situ gels.

### 4.4. Rheological Tests

Polymer-based in situ gels are strongly connected with the residence time in the eye. If the viscosity is low, the preparation stays only very briefly in the eye, while high viscosity might be uncomfortable for the patient. Due to these reasons, it is not enough to measure and evaluate the tested formulations with a viscometer. It is appropriate to rely on rheological parameters, which allow us to analyze the parameters of the produced formulations in detail and properly predict the effects of in situ gels on the eye tissue [74]. Flow curves allowed us to confirm that all of the experimental formulations that we produced were Newtonian fluids, where viscosity does not depend on shear rate [75]. The parameters of dynamic viscosity, when the shear rate is controlled, allow us to compare the behavior of experimental formulations in different temperatures. The results of the tests show that the increase in polymer concentration in the formulations also increased the parameters of dynamic viscosity proportionately with the rise of the temperature. Dewan et al. declared that increased HPMC concentration increased the viscosity of P407 solutions, as well as having a distinct effect on gelation temperatures [76]. Drug release also took longer [76]. Due to this tendency, we can state that gels G5 and G6 do not meet the requirements for ophthalmic preparations, since gels they are prone to gelation even at room temperature. Tests showed that their gelation occurred at 21.5–28.5 °C. It is important for such a composition to stay liquid at room temperature, aiming at its comfortable use. Still, it is important that, having reached the eye’s surface, the formulation would reach the point of gelation, and the preparation would have a longer residence time on the eye’s surface. In our research, we chose HPMC concentrations from 0.5% to 0.75% (*w*/*v*) in our formulations. Cellulose derivatives are also often added to poloxamer forms in order to prolong drug release [77]. HPMC is usually added in 0.5–3% (*w*/*v*) concentrations, depending on molecular mass and poloxamer composition components [35]. Gels G2 and G4 meet the requirements for ophthalmic preparations best of all; their gelation temperature is close to the eye’s surface temperature, which can range from 32.9 °C to 36 °C [48,78]. Formulations G1 and G3 reach the gelation point at higher temperature; therefore, the preparation would have a shorter residence time on the eye surface. Yu et al. wrote that the connection of the hydrogel systems—for example, the ones that contained carboxymethyl chitosan or another natural polymer with poloxamer 407—may possibly improve the biocompatibility of the produced hydrogel [79]. The composition of poloxamer 407 and carboxymethyl chitosan, described by the scientists, had good rheological properties when the gelation temperature was 32–33 °C, and prolonged drug release from hydrogel; this is why this preparation can be considered to be a good, temperature-sensitive ophthalmic drug delivery system [78,79,80]. We used HPMC in our research in order to improve the biocompatibility of in situ gels and enhance the mucoadhesive properties of the preparations. The results of the tests showed that polymer compositions directly influenced the rheological properties of the in situ gels.

### 4.5. Bioactive Componds and Antioxidant Activity of In Situ Gels

When assessing the quality of eye preparations, it is important to determine the quantity of their active ingredients. The drug content of in situ gels G1–G6 was in the range of 94.81–98.37% of the theoretical amounts of phenolic compounds. HPLC test results showed that the bases of the gels were suitable for the introduction of poplar buds extract, because the same compounds were identified in them as in poplar buds extract. Active ingredients were evenly distributed in all formulations. No statistically significant difference (*p* > 0.05) was determined between the amounts of active ingredients in the tested gels. The test results confirmed that the main components in poplar buds are phenolic acids and flavonoids [25]. The results of quantitative analysis showed that in situ gels with poplar buds extract could be used in ophthalmology as sources of polyphenolic compounds [81]. The results of antioxidative activity tests showed that the gels produced with poplar buds extract had antioxidant activity. ABTS radical scavenging tests showed that all of the gels had antioxidant activity statistically significantly lower (*p* > 0.05) than that of the L1 1% aqueous balsam poplar buds extract, used in the in situ gel formulations. The FRAP method also showed statistically significantly lower antioxidant activity than that of the L1 aqueous solution. Though formulations G1–G6 had low antioxidant activity, this was nevertheless significant because of the low concentrations of extract in them. The produced gels can be used as natural sources of antioxidants. Phenolic compounds and flavonoids act as antioxidants [6] and as anti-inflammatory preparations for eye disorders [82].

### 4.6. In Vitro Release Test Determining Total Phenolic Compounds

In vitro diffusion study of the phenolic compounds of the prepared formulations was performed using a Franz diffusion cell [83]. The results of the tests confirmed the data obtained by other scientists—the released amounts of the active compounds depended on the amount of gelling agent [35,46]. The greater the concentration of the gelling agent in the formulation, the slower the rate of release of the active compounds. This confirms previous findings that the amount of active substances released from the formulation depends on its composition [84]. Fathalla et al. reported that the release of ketorolac tromethamine decreases when poloxamer 188 or poloxamer 407 concentration is increased. This decrease in release rate is associated with the increase in the viscosity of the preparation [35]. The results of our research showed that the release of phenolic compounds from prepared in situ gel formulations directly depended on the concentration of gelating polymers. A strong reverse correlation of ρ = −0.991 was determined.

### 4.7. Cell Viability: Short Time Exposure (STE) Test

In situ ocular gels are applied in the treatment of various ophthalmological diseases, and so they must be safe and non-irritating, as they directly affect the eyes. Using SIRC rabbit corneal cells, we performed an eye irritation test: the short time exposure (STE) test. The duration of the test was determined keeping in mind that in situ gels stay longer on the eye’s surface due to their viscosity [85]. SIRC cell viability is defined as the percentage of live cells, as determined via MTT test. After 5 and 30 min exposure and incubation time—which is counted as drug contact with the eye—all of the tested gels had no cytotoxic effects on SIRC cell viability. Asasutjarit et al. demonstrated the safety of a P407/P188/carbopol formulation on a rabbit corneal cell line using short time exposure tests, where the total eye irritation score was zero [86]. Wroblewska et al. described a short time exposure test of biological hydrogel systems on the SIRC cell line. A 0.5% HPMC short time exposure test using MTT dye showed 93.95% viability compared to controls (0.9% solution of sodium chloride) [40]. The results of our tests confirmed the results obtained by other scientists, and showed that the chosen glling agents were suitable for the production of eye drops. The results of the tests also showed that poplar buds extract aqueous solution did not have a cytotoxic effect on the tested cells.

### 4.8. Stability Test

The stability of the medicinal substances in the eye drops depends on their physical and chemical properties, pH, viscosity, excipients, technology, and the chosen packaging [86]. The stability of in situ gels with poplar buds extract was evaluated 30 days after production. The results showed that the physicochemical properties and the total amounts of phenolic compounds of formulations G1–G6 did not change statistically significantly (*p* > 0.05). The formulations were kept in the refrigerator. The formulations remained colorless, clear, and without mechanical impurities. It is possible to conclude that the chosen excipients, their quantity, and their storage conditions determine the physicochemical stability of the formulations. For further research, it is important to choose suitable preservatives and technologies in order to ensure the microbiological stability of the formulations.

## 5. Conclusions

The results of our research showed that the extract of *Populus balsamifera* buds is rich in polyphenols, of which *p*-coumaric acid predominates. Lyophilized poplar buds extract was successfully applied as a source of polyphenols for in situ eye gels, using poloxamer 407 and HPMC. In situ gels’ gelation temperature depends on the concentrations of the polymers poloxamer 407 and HPMC. All of the experimental formulations were characterized by in-situ-gel-specific thermodynamic properties. The sustained release of polyphenols in vitro also depended on the concentrations of the used polymers. The results of this study indicate that the polymer combinations will be expected to be excellent carriers for the prolonged delivery of polyphenols to the surface of the eye. The short time exposure test showed that the experimental formulations did not have an irritating effect on the SIRC cell line. The results of these tests can serve as a basis for further research.

## Figures and Tables

**Table 1:** HPLC analysis elution program. Eluent A: 1% trifluoroacetic acid; eluent B: 100% acetonitrile.

Time, Min		8	30	48	58	65	66	70	71
%A	95	85	80	60	50	50	5	5	95
%B	5	15	20	40	50	50	95	95	5

**Table 2:** Total phenol content and total flavonoids in L1: lyophilized balsam poplar buds extract 1% aqueous solution; and L2: aqueous balsam poplar buds extract.

Extract	Total Phenols mg CAE/g ± SD	Total Flavonoids mg RE/g ± SD
L1	143.44 ± 6.29 ^a^	36.33 ± 1.79 ^a^
L2	88.89 ± 3.27 ^b^	21.12 ± 0.84 ^b^

^a,b^: Series sharing superscript letters showed statistical differences between extracts L1 and L2.

**Table 3:** HPLC analysis of L1: lyophilized 1% aqueous solution of balsam poplar buds extract; and L2: aqueous balsam poplar buds extract (mean mg/g ± SD of dry weight, *n* = 3).

mg/g	L1	L2	Pairwise Difference *
1. Salicin	1.261 ± 0.06	0.618 ± 0.030	ab
2.Chlorogenic acid	0.490 ± 0.03	0.282 ± 0.013	ab
3.Vanillic acid	0.034 ± 0.001	0.012 ± 0.001	ab
4. Caffeic acid	2.569 ± 0.281	1.089 ± 0.050	ab
6. *p*-Coumaric acid	10.720 ± 0.544	6.621 ± 0.284	ab
7. Ferulic acid	0.037 ± 0.001	0.012 ± 0.001	ab
8. Cinnamic acid	4.312 ± 0.198	1.103 ± 0.054	ab
9. Pinobanksin	2.333 ± 0.104	1.627 ± 0.078	ab
10.Apigenin	0.017 ± 0.001	0.004 ± 0.0001	ab
12.Pinocembrin	0.025 ± 0.001	0.008 ± 0.0003	ab
13.Galangin	0.012 ± 0.001	0.003 ± 0.0001	ab
Total flavanoids (mg/g)	2.388	1.642	ab
Total phenolic acids (mg/g)	18.162	9.119	ab
Total amount of identified compounds (mg/g)	21.811	11.379	ab

*: For each active compound, lowercase letters indicate which extracts (a for L1, b for L2) showed a statistically significant pairwise difference.

**Table 4:** Ophthalmic in situ gel compositions, with balsam poplar buds extract.

%*w*/*v*	G0	G1	G2	G3	G4	G5	G6
**L1 (1% aqueous solution extract)**	-	10	10	10	10	10	10
**Poloxamer 407**	15	10	10	12	12	15	15
**HPMC**	0.5	0.5	0.75	0.5	0.75	0.5	0.75
**Propylene glycol**	10	10	10	10	10	10	10
**Purified water**	74.5	69.5	69.25	67.5	67.25	64.5	64.25

**Table 5:** Stability parameters of formulations G1–G6 on day 1 and after 30 days (mean ± SD, n = 3).

In Situ Gels Day 1	Physicochemical Properties and Stability of In Situ Gels					
	**G1**	**G2**	**G3**	**G4**	**G5**	**G6**
Transparency	Clear	Clear	Clear	Clear	Clear	Clear
pH	6.07 ± 0.30	6.29 ± 0.31	6.75 ± 0.33	6.49 ± 0.32	6.15 ± 0.31	6.51 ± 0.33
Refractive index (Water 1.33)	1.355	1.360	1.360	1.360	1.355	1.360
Total phenols mg CAE/g ± SD	13.64 ± 0.66	13.63 ± 0.65	14.02 ± 0.67	14.11 ± 0.69	13.78 ± 0.62	14.06 ± 0.67
**In Situ Gels after 30 Days**						
	**G1**	**G2**	**G3**	**G4**	**G5**	**G6**
Transparency	Clear	Clear	Clear	Clear	Clear	Clear
pH	6.08 ± 0.30	6.33 ± 0.31	6.76 ± 0.33	6.41 ± 0.32	6.18 ± 0.31	6.31 ± 0.32
Refractive index (Water 1.333)	1.360	1.360	1.360	1.360	1.355	1.360
Total Phenols mg CAE/g	13.60 ± 0.64	13.66 ± 0.66	14.11 ± 0.69	14.10 ± 0.68	13.86 ± 0.74	14.09 ± 0.61

**Table 6:** HPLC analysis results for in situ gels G1–G6 (mean µg/g ± SD, n = 3).

Identified Compounds	G1	G2	G3	G4	G5	G6
Galangin (µg/g)	1.17 ± 0.08	1.04 ± 0.05	1.23 ± 0.06	1.24 ± 0.06	1.09 ± 0.05	1.19 ± 0.06
Chlorogenic acid (µg/g)	38.99 ± 1.83	30.61 ± 1.44	31.13 ± 1.46	29.16 ± 1.37	31.24 ± 1.43	30.88 ± 1.38
Caffeic acid (µg/g)	178.79 ± 7.33	171.58 ± 7.37	180.57 ± 7.56	177.14 ± 7.61	182.15 ± 7.47	176.63 ± 8.47
*p*-Coumaric acid (µg/g)	980.18 ± 44.1	971.10 ± 39.81	1035.64 ± 42.43	1077.83 ± 46.31	1017.13 ± 44.74	961.04 ± 41.32
Cinnamic acid (µg/g)	356.72 ± 14.59	335.30 ± 14.07	293.14 ± 12.01	299.45 ± 12.27	312.87 ± 12.16	338.71 ± 12.84
Pinobanksin (µg/g)	221.47 ± 8.61	215.70 ± 8.82	235.87 ± 10.34	241.12 ± 10.37	204.33 ± 8.37	221.05 ± 9.50
Apigenin (µg/g)	1.24 ± 0.05	1.36 ± 0.05	1.49 ± 0.06	1.33 ± 0.05	1.29 ± 0.04	1.49 ± 0.06
Pinocembrin (µg/g)	2.14 ± 0.10	2.06 ± 0.09	2.56 ± 0.12	2.44 ± 0.11	2.03 ± 0.08	2.07 ± 0.07
Total flavanoids (µg/g)	226.02	220.16	241.15	246.13	208.74	225.80
Total phenolic acids (µg/g)	1554.68	1508.59	1540.48	1583.58	1543.39	1507.26
Total amount of active compounds (µg/g)	1780.70	1728.75	1781.63	1829.71	1752.13	1733.06

**Table 7:** Sol-to-gel temperatures of formulations G1–G6 (mean ± SD, *n* = 3).

Formulation	Sol/Gel Temperature ( °C)	Complex Viscosity Pa·s
G1	43.2 ± 1.5	0.0163 ± 0.0005
G2	33.3 ± 1.0	0.0136 ± 0.0004
G3	39.2 ± 1.4	0.0294 ± 0.0009
G4	32.1 ± 1.0	0.0265 ± 0.0008
G5	28.5 ± 0.9	0.0747 ± 0.0023
G6	21.5 ± 0.7	0.0439 ± 0.0013

## Data Availability

All data are available in a publicly accessible repository.
